# Ultrahigh-Pressure Preparation and Catalytic Activity of MOF-Derived Cu Nanoparticles

**DOI:** 10.3390/nano11041040

**Published:** 2021-04-19

**Authors:** Ichiro Yamane, Kota Sato, Ryoichi Otomo, Takashi Yanase, Akira Miura, Taro Nagahama, Yuichi Kamiya, Toshihiro Shimada

**Affiliations:** 1 Graduate School of Chemical Science and Engineering, Hokkaido University, Kita 13 Nishi 8, Kita-ku, Sapporo 060-8628, Japan; ymn_16@eis.hokudai.ac.jp (I.Y.); ah_0403p@eis.hokudai.ac.jp (K.S.); yanase42@eng.hokudai.ac.jp (T.Y.); amiura@eng.hokudai.ac.jp (A.M.); nagahama@eng.hokudai.ac.jp (T.N.); 2 Section of Materials Science, Faculty of Environmental Earth Science, Hokkaido University, Kita 10, Nishi 5, Kita-ku, Sapporo 060-0810, Japan; otomo@ees.hokudai.ac.jp (R.O.); kamiya@ees.hokudai.ac.jp (Y.K.); 3 Division of Applied Chemistry, Faculty of Engineering, Hokkaido University, Kita 13 Nishi 8, Kita-ku, Sapporo 060-8628, Japan

**Keywords:** metal–organic framework (MOF), catalysis, copper nanoparticles, ultrahigh pressure, Huisgen cycloaddition

## Abstract

A metal–organic framework (MOF) consisting of Cu-benzenetricarboxylic acid was processed under ultrahigh pressure (5 GPa) and at temperature of up to 500 °C. The products were characterized with TEM, FTIR, and XAFS. The decomposition of the MOF started at 200 °C at 5 GPa. This temperature was much lower than that in the vacuum. Single-nanometer Cu nanoparticles were obtained in carbon matrix, which was significantly smaller than the Cu particles prepared at ambient pressure. The catalytic activity for Huisgen cycloaddition was examined, and the sample processed at 5 GPa showed a much improved performance compared with that of the MOF-derived Cu nanoparticles prepared without high pressure.

## 1. Introduction

Solid-state copper shows important catalytic functions in organic synthesis [1,2,3,4,5,6,7,8], graphene synthesis [9,10], electrochemical reduction of carbon dioxide [11,12], and purification of automobile exhaust [13,14]. Although group 11 elements in the periodic table show important catalytic activities [15], copper is unique among them because of its natural abundance. For heterogeneous catalysts, nanoparticles (NPs) of active components are desired because a large number of active sites are provided owing to high surface-to-volume ratio in NPs [16,17]. Various methods for the preparation of Cu nanoparticles have been proposed [18]. Among them, metal–organic framework (MOF)-derived metal–carbon composites are recently gathering attention because it is conceptually possible to prepare materials with designed nanostructures [19,20,21,22]. These materials are synthesized by the pyrolysis of MOFs. This method is attractive for the preparation of supported metal NPs because the active site and carrier can be synthesized simultaneously; namely, the metal cations and the ligands in the MOF are converted to metal (or metal-based) NPs and carbon matrix, respectively. However, since the carbonization process involves thermal decomposition of the organic ligands, the products are sensitive to the process parameters. Copper atoms can aggregate to make Cu NPs by pyrolysis because copper does not form stable carbides. However, previous reports show that the pyrolysis of a Cu-containing MOF gives Cu NPs tens of nm [19,20,21,22]. It is desirable to reduce the size of Cu NPs to achieve efficient catalytic functions.

Here, we propose high-pressure and high-temperature (HPHT) synthesis to make metal nanoparticles supported on MOF-derived carbonaceous materials. In general, diffusion in the solid state is expected to be affected by applied pressure. Indeed, in the case of diffusion by vacancy mechanism, it has been reported that the diffusion coefficient decreases with a rise in pressure theoretically and experimentally [23,24,25,26]. Thus, it is expected that pyrolysis under high pressure can prevent nanoparticles from aggregating if their atoms diffused by the vacancy mechanism is dominant.

In this work, we carried out the HPHT treatment of copper benzenetricarboxylic acid (Cu–BTC; C_18_H_6_Cu_3_O_12_) [27,28] to examine the possibility of controlling the carbonization process. The products were characterized by transmission electron microscopy (TEM), X-ray diffraction (XRD), Fourier-transform infrared spectroscopy (FTIR), and X-ray absorption spectroscopy (XAS). We also prepared the samples treated under a vacuum-sealed glass tube for comparison. We found that the precursor Cu-BTC was converted to a copper–carbon composite (Cu@C) at a relatively low temperature, and the samples pyrolyzed sufficiently by the HPHT method contained fine Cu nanoparticles. Finally, we investigated the catalytic activity of the HPHT-treated samples in azide–alkyne Huisgen cycloaddition [5,29,30,31,32].

## 2. Materials and Methods

Cu-BTC, whose trade name is Basolite^®^ C-300, was purchased from Aldrich. Benzylazide, phenylacetylene, 1,4-dioxane, triethylamine, 1,3,5-triisopropylbenzene, and Cu standard solution (1000 ppm aqueous solution) were purchased from FUJIFILM Wako Pure Chemical(Osaka, Japan) and were used without further purification.

High-pressure experiments were carried out using a cubic anvil press (180 ton, CT-factory, Tokyo, Japan) calibrated with phase transitions of bismuth [33]. The Cu-BTC powder was encapsulated in a cylindrical copper capsule, which was embedded in a NaCl filler and then a graphite tube heater, which were then placed in a 13 mm pyrophyllite cube with stainless steel electrodes. We confirmed that the copper capsule was stable and did not interfere with the results. Temperature was measured by an alumel–chromel thermocouple placed in the pyrophyllite cube, which was calibrated with another thermocouple at the sample position in a separate measurement. The sample assembly was compressed to 5 GPa. Then the cell assembly was heated by flowing electric current to the graphite heater in the sample assembly. The temperature increase took approximately 15 min, and the temperature was kept at 200, 300, 400, and 500 °C for 15 min. Then the heating was stopped by switching off the current, and the temperature became below 50 °C only after 30 s. After the sample was completely cooled down, the pressure was released, and the samples were collected from the cubic anvil press under ambient conditions. After grounding by a mortar and pestle, the obtained samples were used for characterization and catalytic reaction.

We synthesized not only the HPHT-treated samples but also the samples pyrolyzed under vacuum. A Cu-BTC pellet was placed in a glass tube with one side closed, and the glass tube was evacuated by a vacuum pump to 6.0 × 10^−1^ Pa. Subsequently, the glass tube was sealed under vacuum by burning off a part of the glass tube with a burner. The Cu-BTC samples sealed in the glass tube were heated in a furnace at different temperatures (i.e., 200, 300, and 400 °C). The temperature was held for 15 min, and then the glass tube was taken out from the furnace. After the samples cooled to room temperature, the sample was collected from the glass tube. The obtained samples were ground into powder by a mortar and pestle and were used for further experiments.

The samples carbonized were analyzed by powder XRD (MiniFlex 600, Rigaku, Tokyo, Japan), FTIR (ICPE-9000, Shimadzu, Kyoto, Japan), and TEM (JEM-2010, JEOL, Tokyo, Japan). Adsorption–desorption isotherms of N_2_ at 77 K were acquired on a Belsorp-mini instrument (BEL Japan Inc., Osaka, Japan). Specific surface areas were estimated by using the Brunauer-Emmett-Teller (BET) equation applied to the adsorption isotherms of N_2_. XAS spectra were taken at BL11S2 of Aichi Synchrotron Radiation Center in transmission mode. The sample and hexagonal boron nitride used as a matrix were mixed with a mortar and pestle for 20 min, and the mixture was pelletized into a 10 mm pellet by using a tablet press for XAS measurement.

**Scheme 1:**
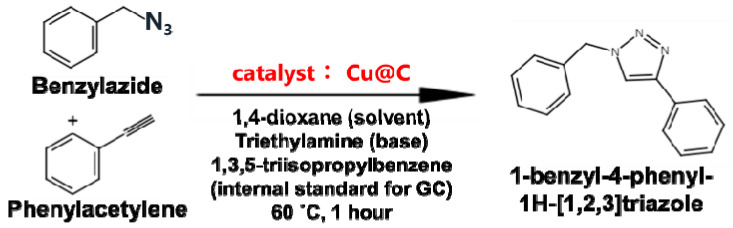
Huisgen cycloaddition for the evaluation of catalytic performance.

The catalytic performance of these samples was evaluated for Huisgen cycloaddition illustrated in Scheme 1 following the procedure in the literature [30]. A powder catalyst (10 mg) prepared as stated above was added to the mixture of benzylazide (0.20 mmol), phenylacetylene (0.22 mmol), triethylamine (0.22 mmol), and 1,4-dioxane (4 mL) in a test tube. Subsequently, a stir bar was added to the test tube, and the tube was sealed by a cap. The reaction solution was heated with stirring at 60 °C for 1 h. Then cycloaddition was quenched by an ice bath. After the catalyst powder was removed from the mixture by filtration, the filtrate was analyzed by gas chromatography (GC) to determine the yield of the cycloaddition product. A gas chromatograph (GC-14B, Shimadzu, Kyoto, Japan) was equipped with a flame ionization detector and capillary column (TC-FFAP, 0.25 mm, 50 m). 1,3,5-Triisopropylbenzene was used as an internal standard. To assign the GC peaks, the filtrate was analyzed with a GC–mass spectrometer (GCMS-QP2010SE, Shimadzu, Kyoto, Japan) equipped with a capillary column (SH-Rxi-5Sil MS, Shimadzu, Kyoto, Japan).

Copper contents in the samples were determined by inductively coupled plasma optical emission spectroscopy (ICP-OES). The samples were completely dissolved in concentrated nitric acid under reflux conditions, and the diluted sample solutions were used for ICP-OES (ICPE-9000, Shimadzu, Kyoto, Japan) measurement. The detail is described in Supplementary Materials.

## 3. Results and Discussion

**Figure 1:**
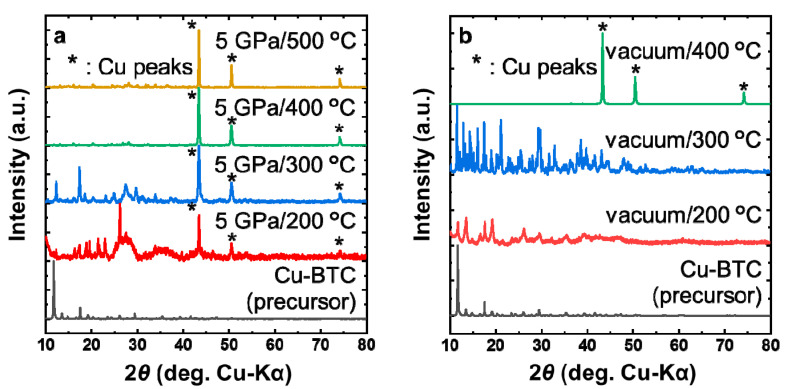
X-ray diffraction patterns of (**a**) the HPHT samples and (**b**) the samples pyrolyzed in the vacuum-sealed glass tubes. The peaks marked with an asterisk (*) at 2*θ* = 43.4°, 50.5°, and 74.1° are assigned to (110), (200), and (220) crystalline planes of metallic Cu, respectively.

The powder XRD patterns and FTIR spectra were acquired to characterize and identify the as-synthesized samples. Figure 1a,b shows the XRD patterns of the MOF-derived samples prepared by the HPHT treatment and pyrolysis in a vacuum-sealed glass tube, respectively. The figure also illustrates that of the precursor Cu-BTC for comparison. As shown in Figure 1a, the three characteristic peaks at 2*θ* = 43.4°, 50.5°, and 74.1° are observed in all HPHT-treated samples, which are absent in the precursor Cu-BTC. These diffraction peaks correspond to (110), (200), and (220) crystalline planes of metallic copper, respectively. No peaks attributed to CuO and Cu_2_O were observed. It is reported that the framework structure of Cu-BTC is robust at room temperature and does not become amorphous below 30 GPa [34]. The appearance of diffraction peaks from Cu indicates that the pyrolysis of Cu-BTC at 5 GPa started at 200 °C or below, resulting in the formation of metallic copper and carbonized BTC framework. Besides, some peaks of the precursor Cu-BTC were maintained in the low-angle region at 5 GPa/200 °C and 5 GPa/300 °C. This suggests that the partly collapsed Cu-BTC framework may remain in these samples. 

On the other hand, the XRD patterns of the samples obtained in the vacuum-sealed glass tube show a different trend from those of the HPHT-treated ones (Figure 1b). For the samples obtained at 200 and 300 °C, the peaks due to not metallic Cu but the partially decomposed precursor Cu-BTC were observed. The peaks became broader, and the intensities significantly changed with temperature increase, indicating that heat treatments decreased the crystallinity with no production of Cu particles. For the sample obtained at 400 °C, three diffraction peaks of metallic Cu were observed as in the case of HPHT-treated samples. Therefore, it is apparent that the Cu-BTC pyrolyzed and carbonized at 400 °C in the vacuum-sealed glass tube. 

**Figure 2:**
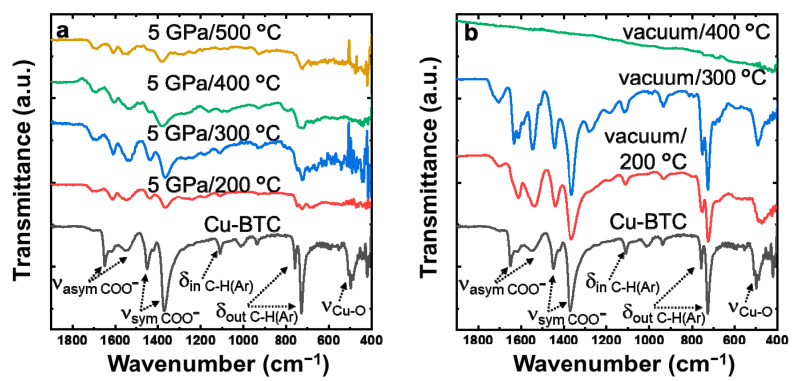
FTIR spectra of (**a**) the HPHT samples and (**b**) the samples pyrolyzed in the vacuum-sealed glass tubes. Marks of IR peaks ν, δ, and Ar in the figures denote stretching, bending, and the benzene ring, respectively.

Figure 2a,b shows the IR spectra of the HPHT-treated samples and those heated in a vacuum-sealed glass tube, respectively. The IR spectrum of the precursor Cu-BTC is given for comparison. Several characteristic bands are observed in the spectrum of the precursor Cu-BTC. The band at 486 cm^−1^ corresponds to the Cu–O stretching vibration. The two bands at 730 and 760 cm^−1^ correspond to the out-of-plane C–H bending vibration of the benzene rings of the Cu-BTC, while the band at 1110 cm^−1^ corresponds to their in-plane C–H bending vibration. The two bands at 1373/1449 cm^−1^ and 1571/1630 cm^−1^ represent the symmetric and asymmetric stretching vibration of the carboxylate groups, respectively [34]. For the spectra of all the HPHT-treated samples, the Cu–O band disappears as shown in Figure 2a, which means that Cu^2+^ no longer coordinated with carboxyl groups COO^−^ and the crystal structure of Cu-BTC was collapsed. The bands corresponding to the benzene rings and carboxylate groups gradually disappeared as the temperature was increased. This indicates that the pyrolysis of the BTC (i.e., C_6_H_3_(COO^−^)_3_) proceeded at temperatures higher than that for metallic Cu formation. It is considered that the benzene ring decomposed to carbonaceous substances, and COO^−^ was eliminated as CO_2_. This decarboxylation was associated with the reduction of Cu^2+^ to metallic Cu. This is consistent with the results of XRD. Besides, the IR spectra indicate that a small amount of benzene ring and carboxy groups remained in the samples treated at 400 and 500 °C under 5 GPa, although their XRD patterns exhibited only metallic Cu peaks. It means that the carbonized matrix was amorphous.

The samples heated at 200 and 300 °C in the vacuum-sealed glass tube had IR bands similar to those of the precursor Cu-BTC, while no such bands were observed in the sample obtained at 400 °C. This result indicates that the Cu-BTC was not pyrolyzed at 300 °C or below, while the benzene ring was fully converted to carbon materials by pyrolysis at 400 °C. This is also consistent with the result of XRD.

The copper content of the synthesized samples was examined by ICP-OES. As shown in Table 1, the relative Cu content increased with an increase in the treatment temperature for the HPHT-treated samples. It reflects the progress of pyrolysis associated with the loss of carbon species, which is consistent with the results of XRD and FTIR. The Cu contents of 5 GPa/200 °C and 5 GPa/300 °C samples were lower than that of the precursor Cu-BTC (31.5 wt%), and this may be caused by moisture absorption on the samples. On the other hand, for the samples obtained in the vacuum-sealed glass tube, the Cu content of the unpyrolyzed ones (<300 °C) was almost the same as that of the Cu-BTC. Subsequently, the Cu content was increased drastically by heating at 400 °C, indicating the occurrence of the pyrolyzation.

Previous studies have reported that Cu-BTC decomposes at >400 °C under ambient pressure [35,36,37,38]. Our experiments show that heating of Cu-BTC in vacuum had the same trend. However, under high pressure (5 GPa), Cu-BTC started to decompose and produce metallic copper at a lower temperature than those conditions. This suggests that the high pressure shifts the equilibrium to the side to decompose Cu-BTC to produce Cu. 

**Figure 3:**
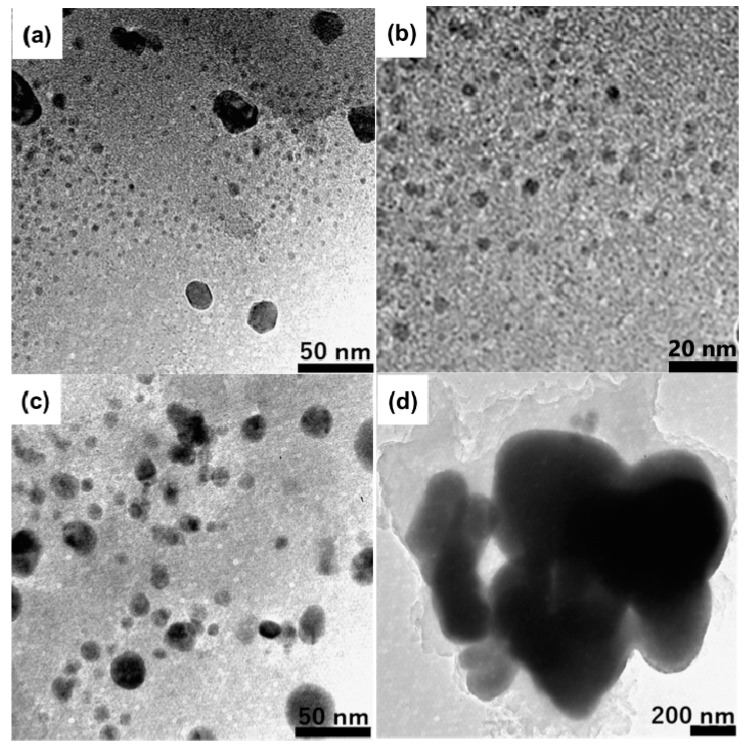
TEM images of (**a**) Cu@C synthesized at 5 GPa/500 °C, (**b**) magnified view of (**a**), (**c**) Cu@C synthesized at 5 GPa/400 °C, and (**d**) Cu@C synthesized in a vacuum-sealed glass tube (vacuum/400 °C).

The morphology of Cu particles in the samples was examined by TEM. As shown in Figure 3, the samples sufficiently pyrolyzed contain Cu as NPs in the carbon matrix. For the samples synthesized at 5 GPa/500 °C and 5 GPa/400 °C, the sizes of Cu NPs are around 5 and 10 nm, respectively, which are smaller than that pyrolyzed in a vacuum-sealed glass tube. Furthermore, the Cu NPs are also smaller than the values of the reported Cu-BTC-derived materials pyrolyzed at ambient pressure [35,36,37]. Therefore, high-pressure treatments enable the Cu NPs to prevent from sintering without any special templates or support materials. This pressure effect can be originated from the pressure dependency of the diffusion coefficient.

**Figure 4:**
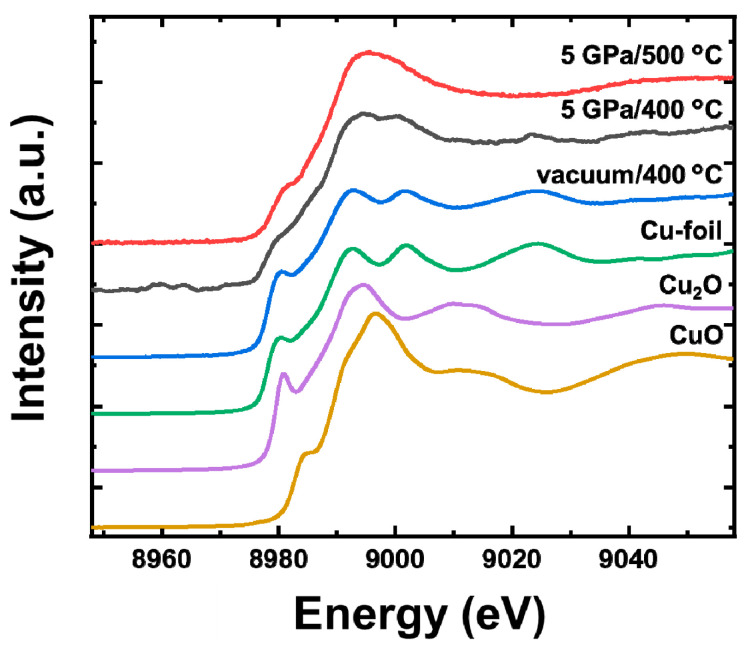
XANES spectra of MOF-derived Cu@C and reference samples.

The samples pyrolyzed sufficiently were further characterized by X-ray absorption near edge structure (XANES) in order to evaluate the valence state of Cu in the NPs. The normalized XANES spectra of these samples and references (Cu-foil, Cu_2_O, and CuO) are shown in Figure 4. The sample synthesized in the vacuum-sealed glass tube at 400 °C showed a similar XANES spectrum to that of the Cu-foil in the spectrum, whereas the samples prepared at 5 GPa/400 °C and 5 GPa/500 °C were quite different from those of the references. The main absorption peak of the sample prepared at 5 GPa slightly shifted toward higher energy, and the slope on the rising of the peak became smaller than that of the Cu-foil. It is in contrast to the fact that only metal Cu peaks were observed in the XRD patterns of all these samples. This indicates that the spectra of the HPHT-treated samples contained the component for those of Cu_2_O and CuO. Thus, it is revealed that the surface of Cu NPs in HPHT-treated samples was partially oxidized. The XANES spectra were curve-fitted by linear combination of the spectra of the Cu-foil, Cu_2_O, and CuO. The result is shown in Table 2 and Figure S1. The detail of the curve fitting is shown in Supporting Information.

The porous nature of the carbonaceous matrix in these samples was characterized by N_2_ adsorption measurements, with the specific surface areas (SSAs) calculated by the BET method. The results are shown in Table 3. Only the one synthesized at 200 °C had mesopores as in the precursor Cu-BTC, while the other ones showed macropores. The rather complicated behavior of the SSAs shown in Table 3 can be explained as follows. In the vacuum-treated samples, the SSAs decreased significantly by the deterioration in crystallinity at 200 and 300 °C. At 400 °C, the pyrolysis proceeded, and CO_2_ and other volatile species were formed, resulting in channels in the products and in an increase in the SSAs. In the HPHT-treated samples, very small SSAs at 200 °C can be explained by the collapse of the pores of a MOF by decomposition under pressure. The increase of SSAs from 300 to 400 °C can be explained by the pores created by trails of Cu NPs during the aggregation. At 500 °C, the Cu aggregation is hindered by the quick carbonization of the MOF ligand, thus preventing the formation of long connected channels.

Huisgen cycloaddition is one of the most representative click reactions that produce 1,2,3-triazole and is accelerated by Cu catalysts [18,30]. The yields of the product in Huisgen cycloaddition are shown in Table 4. 

The HPHT pyrolyzed samples exhibited catalytic activity, which was improved with a rise in treatment temperature. On the other hand, vacuum-pyrolyzed samples did not show catalytic activity. We can consider three factors governing the catalytic activity in this case. The first is the exposure of the Cu NP surface to the outside. If the carbon species cover the Cu NPs, the Cu NPs do not work as catalysts. Under a confined environment, the decomposed species may cover the surface of the Cu NPs. This explains the result in which the samples obtained in the vacuum-sealed glass tube did not show catalytic activity. In contrast, the HPHT environment was in the air, and carbonaceous matrix species covering the Cu NP can be partially combusted. Actually, we can see some small gaps between the Cu NPs and the surrounding matrix in the TEM images of the HPHT-treated samples (Figure 3a–c), whereas the samples treated in the vacuum-sealed glass tube were totally covered by carbon matrix (Figure 3d). 

The second factor is the surface-to-volume ratio of the Cu NPs. In general, Cu NPs easily sinter and aggregate at >300 °C, leading to a decrease in catalytic activity. In this work, however, we succeeded in inhibiting the diffusion of Cu atoms by treating them under high pressure, thus preventing the aggregation. This effect makes it possible to increase the relative copper loading ratio contained in the samples without sintering the particles, which we consider to be responsible for the increase in the catalytic activity of the HPHT samples treated at higher temperatures. 

The third factor is the oxidation state of the NP surface. It has been reported that not only CuI salts but also mixed Cu/Cu–oxide NPs or CuO can act as catalysts [1,5,30,39]. The XANES results showed that the surfaces of the Cu NPs in the samples synthesized at 5 GPa/400 °C and 5 GPa/500 °C were partly oxidized, forming mixed Cu/Cu–oxide NPs. The 5 GPa/500 °C sample was more oxidized. These results are in accordance with the catalytic activity. The high oxidation ratio of the HPHT 500 °C sample is explained by the high surface-to-volume ratio of the smallest Cu NPs among the samples.

The second and third factors come from the small size of the Cu NPs, which originated from the inhibited diffusion of Cu atoms at a high-pressure condition during the decomposition of the MOF. The present result offers a strategy for the preparation of highly active catalysts based on metal nanoparticles synthesized from the decomposition of the MOF and metal complex-based materials.

## 4. Conclusions

We examined the HPHT treatment of the Cu-containing MOF (Cu-BTC) to prepare Cu NPs supported on the carbonaceous material. The aggregation of Cu NPs was prevented, and sub-10 nm NPs were obtained. It was found that the decomposition temperature of Cu-BTC was lowered from 400 °C at ambient pressure or vacuum to 200 °C at 5 GPa. The Cu@C samples prepared by the HPHT treatment showed high activity for Huisgen cycloaddition reaction.

## Figures and Tables

**Table 1:** Cu content determined by ICP-OES of MOF-derived Cu@C materials.

Scheme	Cu Content
5 GPa/500 °C	63 wt%
5 GPa/400 °C	56 wt%
5 GPa/300 °C	27 wt%
5 GPa/200 °C	25 wt%
Vacuum/400 °C	54 wt%
Vacuum/300 °C	30 wt%
Vacuum/200 °C	30 wt%

**Table 2:** XANES analysis for Cu valency of MOF-derived Cu@C materials.

Synthesis Conditions	Cu	Cu_2_O	CuO
	Atomic %	Atomic %	Atomic %
5 GPa/500 °C	26.2 ± 1.9	22.6 ± 2.3	51.2 ±4.5
5 GPa/400 °C	44.9 ± 4.8	31.9 ± 5.7	23.2 ± 8.3
Vacuum/400 °C	79.3 ± 1.4	20.7 ± 3.0	0.0 ± 0.9

**Table 3:** Surface areas measured by N_2_ adsorption and the BET method of MOF-derived Cu@C materials, pristine Cu-BTC.

Synthesis Conditions of Catalysts	Surface Area (m^2^g^−1^)
5 GPa/500 °C	6.7
5 GPa/400 °C	22.6
5 GPa/300 °C	9.1
5 GPa/200 °C	0.4
Vacuum/400 °C	13.6
Vacuum/300 °C	9.7
Vacuum/200 °C	124.6
Cu-BTC	937.6

**Table 4:** Yields of the product in Huisgen cycloaddition over by MOF-derived Cu@C catalysts.

Synthesis Conditions of Catalysts	Yield
5 GPa/500 °C	28%
5 GPa/400 °C	18%
5 GPa/300 °C	16%
5 GPa/200 °C	0%
Vacuum/400 °C	0%
Vacuum/300 °C	0%
Vacuum/200 °C	0%
Blank (no catalyst)	0%

## Data Availability

The data is available on reasonable request from the corresponding author.

## Supplementary Materials

The following are available online at https://www.mdpi.com/article/10.3390/nano11041040/s1, 1. Preparation of ICP-OES Samples; 2. (Figure S1) Curve Fitting of XANES spectra.
