# Supplementary material for: Ultrahigh-Pressure Preparation and Catalytic Activity of MOF-Derived Cu Nanoparticles
Source: Nanomaterials (Basel). 2021 Apr 19;11(4):1040. doi: 10.3390/nano11041040 (PMC8073665; doi:10.3390/nano11041040)
Supplement: Supplementary file 1 [file nanomaterials-11-01040-s001.zip › nanomaterials-1173131-supplementary.pdf]

Supporting Information

Ultrahigh pressure preparation and catalytic activity of MOF-derived Cu nanoparticles

Ichiro Yamane[1], Kota Stato[1], Ryoichi Otomo[2], Takashi Yanase[3], Akira Miura[3], Taro Nagahama[3], Yuichi Kamiya[2], Toshihiro Shimada[3]

1 Graduate School of Chemical Science and Engineering, Hokkaido University, Kita 13 Nishi 8, Kita-ku, Sapporo, 060-8628 Japan

2 Division of Environmental Materials Science, Graduate School of Environmental Science, Hokkaido University, Kita 10, Nishi 5, Kita-ku, Sapporo, 060-0810 Japan

3 Division of Applied Chemistry, Faculty of Engineering, Hokkaido University, Kita 13 Nishi 8, Kita-ku, Sapporo, 060-8628 Japan

1. Preparation of ICP-OES Samples

Cu standard solution (Cu 1000 ppm, solvent 0.1 mol/L HNO_3) was diluted with 1.3 mol/L HNO_3 to prepare Cu standard samples for making calibration curve at concentrations of 10 ppm, 5 ppm, 2.5 ppm, 1.25 ppm, and 500 ppb. Sample solutions to measure Cu content were prepared as follows: the sample powder was added to a flask with 13 mol/L (60 wt%) HNO_3 10 mL, then the dispersion was heated until all of the powder was dissolved with refluxing. The obtained solutions were diluted with distilled water to make the HNO_3 concentration of 1.3 mol/L. These original solutions were further diluted with 1.3 mol/L HNO_3 to prepare two sample solutions with different concentrations for one sample.

2. Curve Fitting of XANES spectra

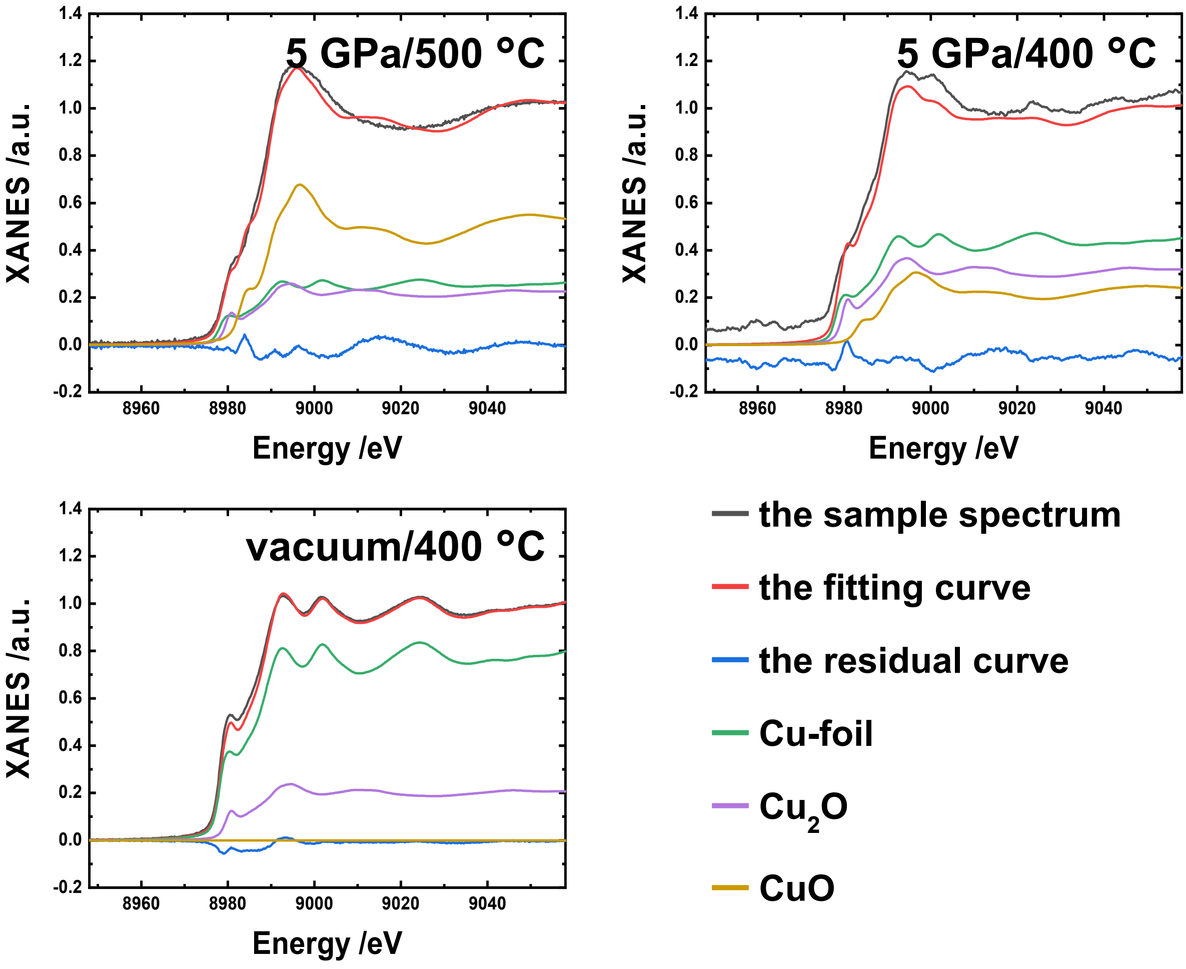

Figure S1: Curve fitting of the XANES spectra was least square of the residuals using spectra of Cu foil, Cu_2O and CuO.
